# Numerical simulation of efficient fully 2D-stacked perovskite photovoltaics using Ruddlesden–Popper/dichalcogenide heterojunctions

**DOI:** 10.1039/d6ra06588k

**Published:** 2026-09-01

**Authors:** Mustafa Kareem, Mustafa Abdullah, Praharshkumar B. Raj, Badri Narayan Sahu, Sridharan Sundharam, Sanjeev Kumar

**Affiliations:** a College of Remote Sensing and Geophysics, Al-Karkh University of Science Haifa St. Baghdad 10011 Iraq dr.mustafa@kus.edu.iq; b College of Science, University of Warith Al-Anbiyaa 56001 Karbala Iraq; c Hourani Center for Applied Scientific Research, Faculty of Engineering, Al-Ahliyya Amman University Amman Jordan; d Department of Chemistry, Faculty of Science, Gokul Global University Sidhpur Gujarat India; e Department of Electronics & Communication Engineering, Siksha 'O' Anusandhan (Deemed to be University) Bhubaneswar Odisha-751030 India; f Department of Physics, Sathyabama Institute of Science and Technology Chennai Tamil Nadu India; g Department of Physics, Institute of Sciences, Chandigarh University Mohali Punjab India

## Abstract

With the scaling trends in photovoltaics moving toward thinner photoactive layers, atomically thin two-dimensional (2D) materials are emerging as a prime option for coupling with next-generation solar cells. We report a heterostructure of an all-2D Ruddlesden–Popper perovskite solar cell (PSC) incorporating two transition metal dichalcogenides (TMDs), molybdenum disulfide (MoS_2_) and tungsten disulfide (WS_2_), as charge-transporting layers. The proposed MoS_2_/2DRP/WS_2_ heterostructure provides favorable energy-level alignment and efficient charge-selective transport, resulting in enhanced device efficiency. We analyze the effects of perovskite layer thickness, trap-state density, charge-carrier mobility, operating temperature, and parasitic resistances on the photovoltaic parameters. After optimization using experimentally supported material properties and reliable interface defect densities, the proposed PSC achieved a simulated power conversion efficiency (PCE) of 25.249%, demonstrating the potential of 2D-stacked RP/TMD heterostructures.

## Abstract 2

This article reports a heterostructure of an all-2D Ruddlesden–Popper perovskite solar cell incorporating two dichalcogenides as charge-transporting layers to achieve a PCE of 25.249%

## Graphical abstract

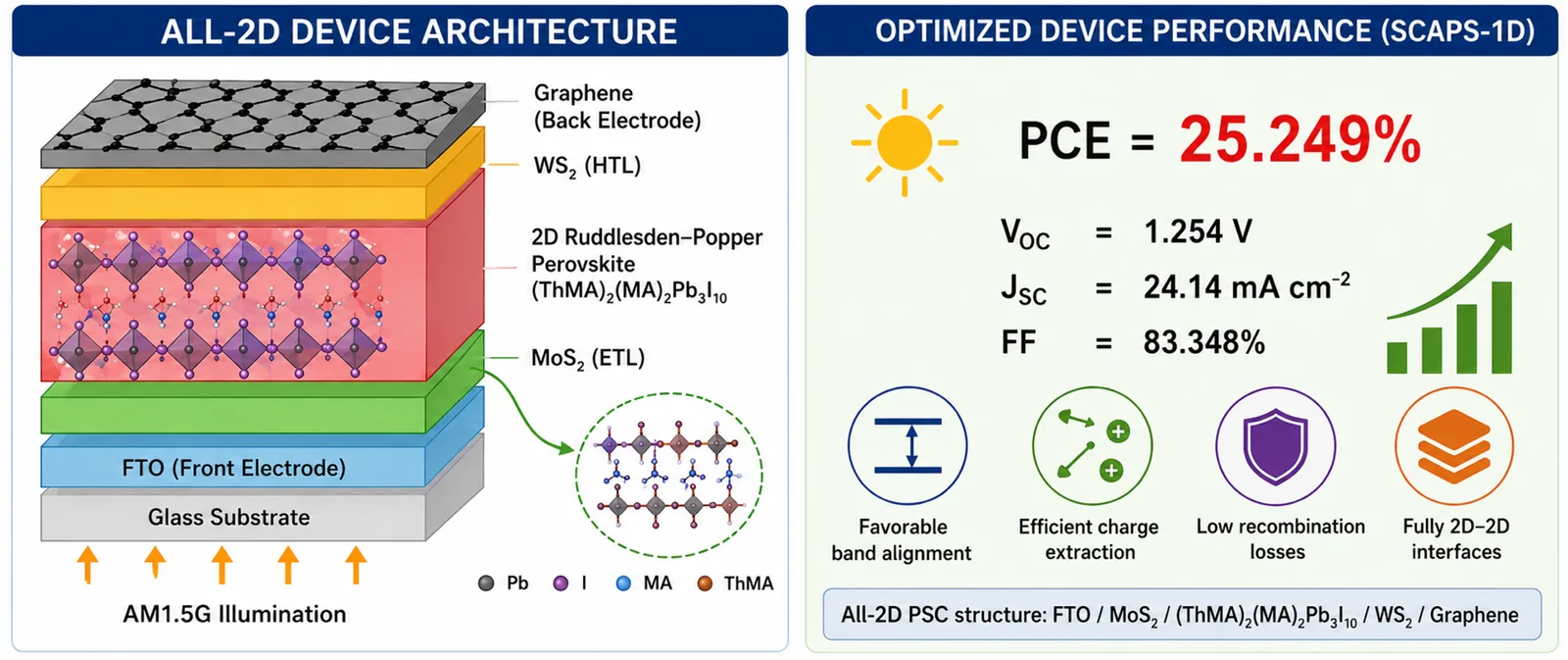

## 1. Introduction

The development of advanced power-generation technologies that are lightweight, structurally simple, and capable of long-term operation without external fuel is necessary to provide a sustainable energy supply under harsh conditions.^1^ Among all, solar cells offer a promising and environmentally viable technique for renewable energy generation.^2^ Two-dimensional Ruddlesden–Popper (2DRP) perovskites have gained significant interest for utilization in solar cells owing to their enhanced moisture and heat tolerance, markedly improved photostability, minimal self-doping (mitigating unavoidable traps), and considerably lower ion mobility compared to 3D perovskites (due to quantum-well orientation).^3,4^ Bulky organic cations separate the inorganic sheets through electrostatic and hydrogen bonding, giving structural rigidity.^5^ The Goldschmidt tolerance factor and octahedral factor as structural parameters have been widely used to evaluate the geometric fit and structural characteristics of the perovskite frameworks and provide helpful insights for understanding the stability of different perovskite structures.^6^ Moreover, the 2DRP layered architecture allows for higher bandgap tuning and material optimization.^7^ However, the relatively low efficiency, which is mainly caused by interfacial defects, prevents their practical applications.

2D materials such as graphene, MXenes, and chalcogenides have been recognized as promising candidates for interface engineering thanks to their lack of dangling bonds, layer-dependent electronic properties, adjustable functional groups, and intrinsic compactness.^8–10^ In these 2D nanomaterials, the atoms are tightly bound *via* robust in-plane covalent bonds forming layers, while the atomically thin layers are arranged vertically by weak van der Waals forces to create a 3D lattice.^11,12^ Therefore, the ultralow thickness makes these nanomaterials naturally flexible, lightweight, and semi-transparent.^13^ Transitional metal dichalcogenides (TMDs) are a rising star in the post-graphene era, consisting of two chalcogens and a transition metal. Molybdenum disulfide (MoS_2_) and tungsten disulfide (WS_2_) are two well-known and extensively explored TMDs because of their tunable band structure configured for various optoelectronics.^14^

Perovskite films can be deposited on the MoS_2_ by using appropriate interface engineering and surface treatments.^14^ Studies revealed that chemical vapor deposition (CVD) method could be suitable for fabricating pinholes-free and ultrathin MoS_2_ transport layers, while alternative approaches such as solution processing or sputtering have also been reported for TMD thin films.^15^ Because pristine MoS_2_ is relatively inert and hydrophobic, achieving uniform growth of the perovskite film may require surface modifications, such as UV-ozone or oxygen plasma treatment, to enhance surface energy and precursor wettability.^16^ Recent advances in TMD integration and interface engineering indicate that the proposed architecture is experimentally feasible, although optimization of the deposition process and interface quality remains an important direction for future work.^17^ This opens up multiple possibilities to design vertical heterostructures for PSCs without the lattice mismatch issue in traditional heterostructures. Thus, numerical investigations of fully 2D–2D heterojunctions are expected to enable optimization of 2DRP perovskite material. Herein, we report simulation of fully 2D layered perovskite photovoltaics using 1D Solar Cell Capacitance Simulator (SCAPS-1D) software. Although 2DRP perovskites and TMD-based charge-transport materials have been studied separately previously, systematic integration and numerical optimization of 2DRP with MoS_2_ and WS_2_ in an all-2D fluorine-doped tin oxide (FTO)/MoS_2_/2DRP/WS_2_/graphene architecture are not sufficiently investigated. This work presents (ThMA)_2_(MA)_*n*−1_Pb_*n*_I_3*n*+1_ (nominal *n* = 3) RP structure as a light harvester layer, which provides a layered framework using a 2-thiophenemethylammonium (ThMA^+^) organic spacer with a relatively extended inorganic framework for charge transport. Briefly, the 2DRP perovskite was sandwiched between the WS_2_ hole-transporting layer (HTL) and the MoS_2_ electron-transporting layer (ETL). The optimized 2DRP perovskite absorber enabled thin 2D PSCs with a low trap state density and allowed effective charge transfer. An efficient fully PSC with a PCE up to 25.249% was achieved with an open circuit voltage (*V*_OC_) of 1.254 V, a short-circuit current density (*J*_SC_) of 24.14 mA cm^−2^, and a fill factor (FF) of 83.348%. The 2DRP PSCs retained about 93% of their original PCE during temperature-dependent electrical performance analysis up to 95 °C.

## 2. Methodology

PSCs can be numerically computed using the SCAPS-1D software, which was developed by Burgelman *et al.*^18^ We used the SCAPS-1D tool owing to its ability to analyze and optimize the photoelectric properties of the proposed 2D solar cell structure utilizing a 1D drift-diffusion framework.^19^ This simulation program can be utilized to evaluate energy band structure, incident-photon-to-current efficiency (IPCE), charge-carrier transfer dynamics, recombination rate, current–voltage (*J*–*V*) properties, Mott–Schottky characteristics, and impedance spectroscopy simulations under AC perturbations.^20^ Under illumination conditions, the semiconductor solar cells can be explained through the continuity equation for charge carriers with the proper boundary specifications through the simultaneous solution and the Poisson equation. The charge-carrier dynamics are studied *via* the drift-diffusion mode, which is stated in terms of the quasi-Fermi level. Recombination mechanisms considered include radiative, Auger, and Shockley-Read-Hall (SRH) recombination. The diffusion length was calculated using the Einstein relation 
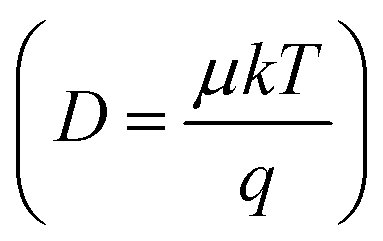
 and the carrier lifetime extracted from the SRH recombination model implemented in SCAPS-1D 
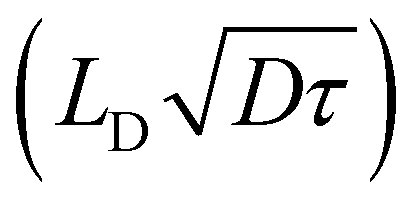
. Table 1 lists the key parameters significant for the PSC simulation, adopted from literature, while Table 2 describes interfacial defects at ETL/perovskite and perovskite/HTL heterojunctions. The PSCs were simulated under the AM1.5 illumination spectrum of 1000 W m^−2^ at an operating temperature of 25 °C and a frequency of 1.0 MHz. In all layers, the thermal velocities of the electrons and holes are kept constant at 10^7^ cm s^−1^. To ensure more reliable and realistic device simulations, an interface defect density of 10^12^ cm^−2^ was considered, along with a series resistance (*R*_S_) of 4.0 ohm cm^2^ and a shunt resistance (*R*_SH_) of 1000 ohm cm^2^. However, time-dependent degradation processes in the active and charge transport layers, such as chemical changes, thermal exposure duration, and ionic migration, cannot be captured by SCAPS-1D because it operates under steady-state conditions. Besides, this simulation focuses on a drift-diffusion-based photoelectric evaluation of the PSC architecture, utilizing experimentally reported parameters, rather than a microscopic exploration in 2D materials, such as excitonic effects, quantum confinement, and interface dipoles.

**Table 1:** The literature-based parameters of the layers utilized in the initial PSC simulation

Parameters	MoS_2_	(ThMA)_2_(MA)_2_Pb_3_I_10_	WS_2_
Thickness (nm)	3.0	450	3.0
*E*_g_ (eV)	2.3	1.59	2.0
*χ*_e_ (eV)	4.2	3.95	3.75
*ε*_r_	4.0	25	6.0
*N*_C_ (cm^−3^)	7.5 × 10^17^	2.2 × 10^18^	1.0 × 10^18^
*N*_V_ (cm^−3^)	1.8 × 10^18^	1.8 × 10^19^	5.0 × 10^17^
*µ*_e_ (cm^2^ V^−1^ s^−1^)	40	1.76 × 10^−3^	52
*µ*_h_ (cm^2^ V^−1^ s^−1^)	26	1.78 × 10^−3^	52
*N*_D_ (cm^−3^)	8.0 × 10^17^	—	—
*N*_A_ (cm^−3^)	—	1.0 × 10^16^	3.87 × 10^18^
*N*_T_ (cm^−3^)	9.0 ×10^18^	5.0 × 10^16^	1.5 × 10^17^
References	21–24	25	26–31

**Table 2:** Interface parameters of PSC

Parameters/interfaces	MoS_2_/(ThMA)_2_(MA)_2_Pb_3_I_10_	(ThMA)_2_(MA)_2_Pb_3_I_10_/graphene
Defect type	Neutral	Neutral
Capture cross section for electrons (cm^2^)	1 × 10^−19^	1 × 10^−19^
Capture cross section for holes (cm^2^)	1 × 10^−19^	1 × 10^−19^
Energetic distribution	Single	Single
Reference for defect energy level *E*_t_	Above the highest *E*_V_	Above the highest *E*_V_
Energy with respect to reference (eV)	0.600	0.600
Total defect density (cm^−2^)	1.0 × 10^12^	1.0 × 10^12^

## 3. Results and discussion

**Fig. 1:**
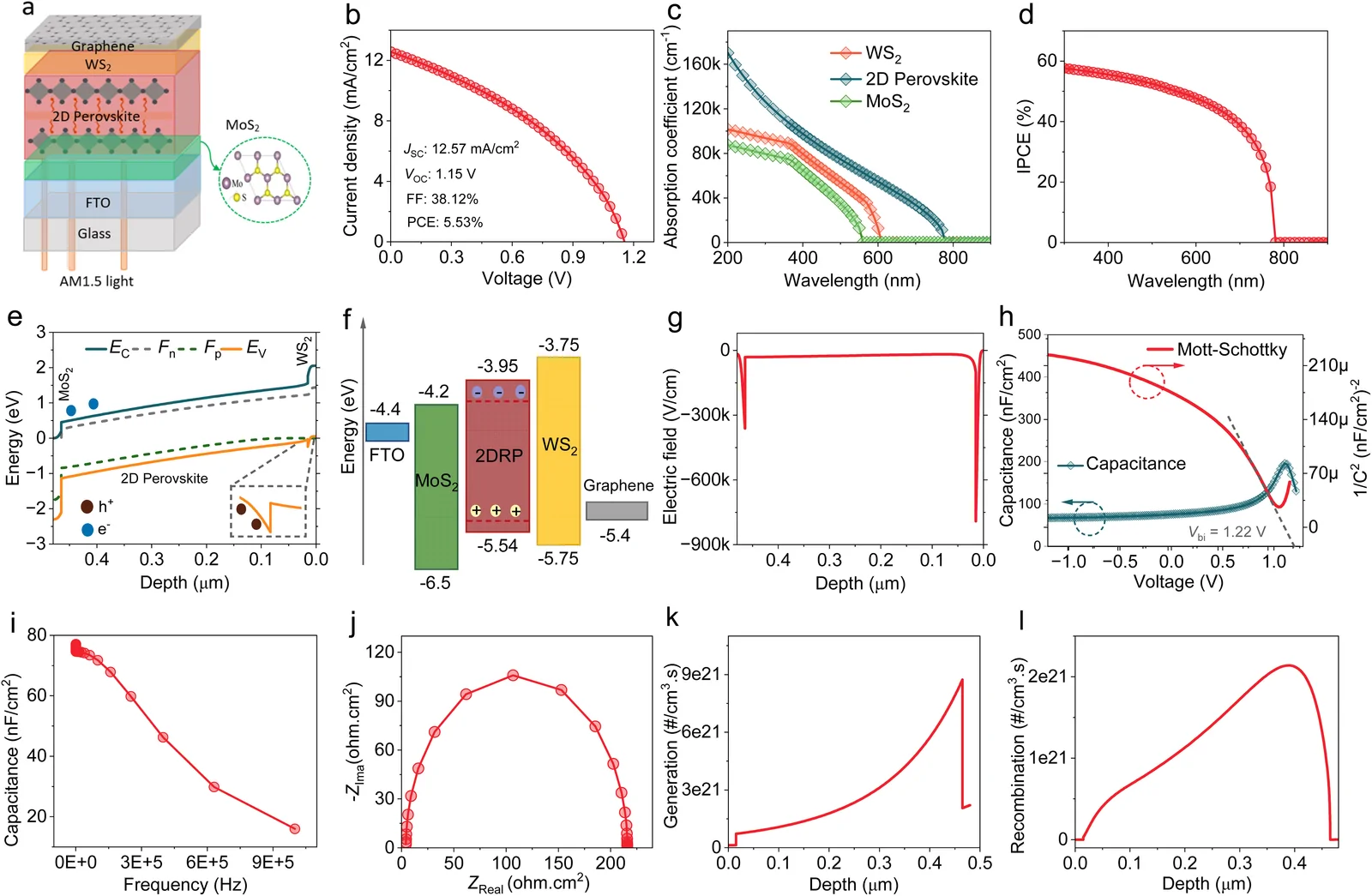
Device schematic and initial SCAPS-1D simulation outcomes for the designed PSC structure. (a) Structure of the 2D-stacked solar cell. (b) *J*–*V* plot of PSC under AM1.5 illumination. (c) Absorption spectra of MoS_2_, WS_2_, and 2DRP layers. (d) IPCE curve of PSC. (e) Energy band alignment of MoS_2_/(ThMA)_2_(MA)_2_Pb_3_I_10_/WS_2_. Inset: the valence band cliff at perovskite/ETL. (f) Energy levels of all PSC layers. (g) Electric field distribution across the PSC. (h) *C*–*V* and Mott–Schottky plot under dark conditions. (i) *C*–*f* characteristic of PSC under AC bias. (j) Nyquist plot. (k) Charge-carrier generation. (l) Total recombination pattern.

Fig. 1a shows the schematic structure of a 2D-layered solar cell employing 2D Ruddlesden–Popper perovskite as a light absorber material. The FTO substrate was chosen for its high optical transparency and electrical conductivity, suitable for light entry and electron collection. MoS_2_ and WS_2_ TMDs were selected as the charge transport layer due to their appropriate electronic properties for carrier extraction and compact interface with the perovskite absorber. While, graphene was added as a back electrode due to its mechanical flexibility and compatibility with an all-2D photovoltaic architecture. The stacked FTO/MoS_2_/2DRP/WS_2_/graphene configuration ensures effective charge separation and collection and limited recombination losses across each interface. Fig. 1b illustrates the *J*–*V* curve of the simulated PSC under AM1.5 light. Based on initial input parameters, the device attains a PCE of 5.53% with poor FF and *J*_SC_. The absorption coefficient in Fig. 1c is derived from 
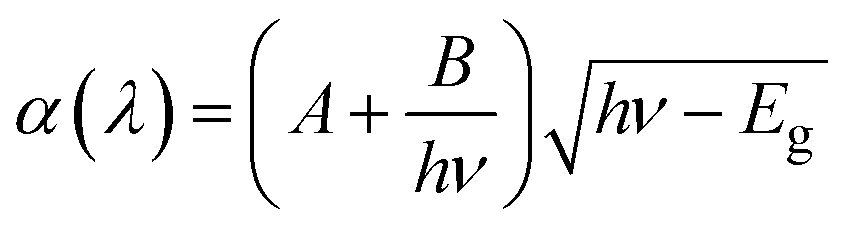
 and confirms the photoconversion abilities of the individual layers, with the 2DRP layer revealing the highest spectrum. As exhibited in Fig. 1d, the device showed a low IPCE response for visible light that aligns with the low *J*_SC_ trend. The suitable band alignment and energy level offsets at the interfaces are shown in Fig. 1e and f, facilitating charge carrier drifting, thus improving *V*_OC_. Fig. 1g demonstrates electric field distribution across the device, revealing a strong electric field at interfaces. This behaviour is corroborated by the Mott–Schottky analysis (Fig. 1h), revealing a high built-in potential (*V*_bi_) of 1.22 V, which exceeds the corresponding *V*_OC_ (1.15 V), confirming the physical consistency of the simulated junction prior to device optimization. Fig. 1i exhibits the *C*–*f* curve of the PSC under AC bias, utilizing it to extract the Nyquist plot. The impedance modeling shows a low recombination resistance (*R*_rec_), indicating enhanced interfacial recombination, which contributes to the reduction in the FF. The low charge-carrier extraction is further confirmed by generation–recombination profiles (Fig. 1k and l).

**Fig. 2:**
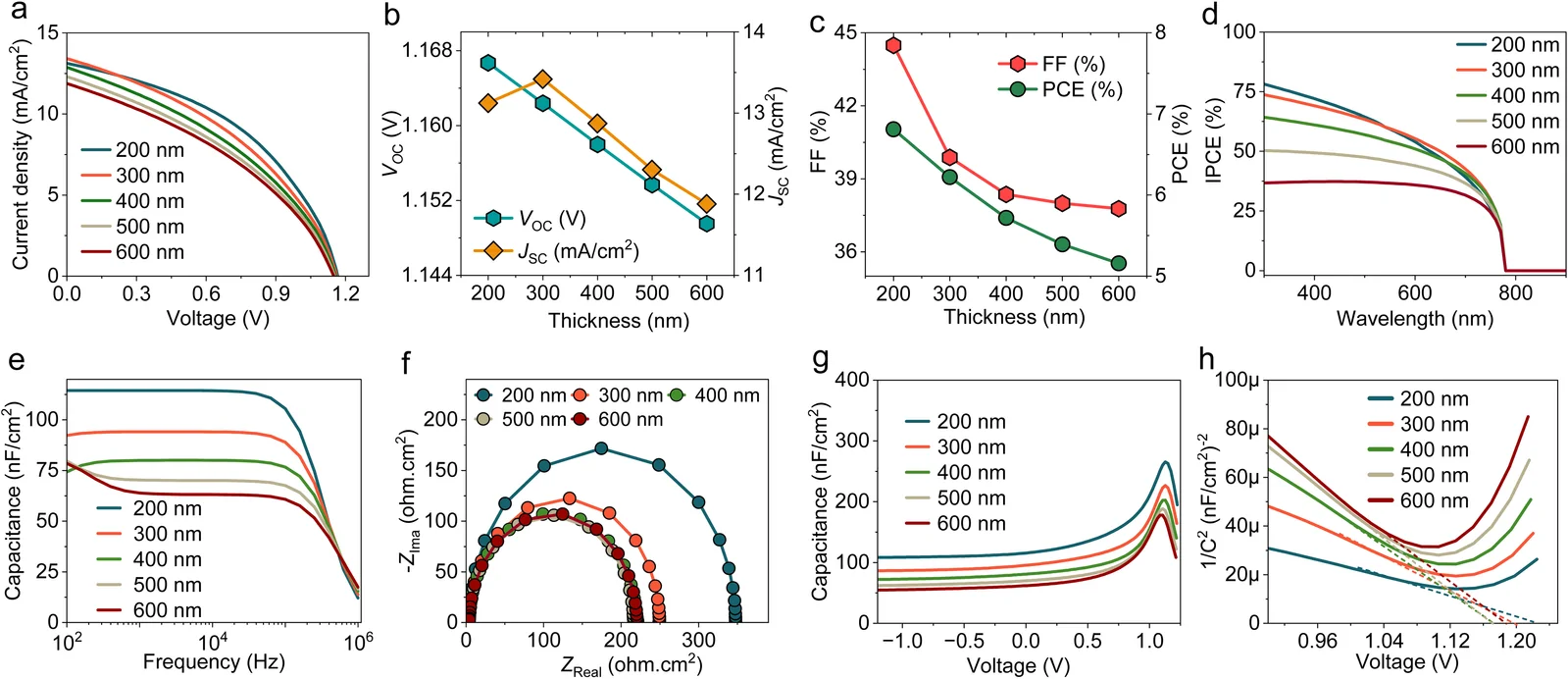
Effect of perovskite absorber thickness on the performance of PSCs. (a) *J*–*V* plots. (b) Dependency of *V*_OC_ and *J*_SC_ on perovskite thickness. (c) Evaluation of FF and PCE with perovskite thickness. (d) IPCE spectra. (e) *C*–*f* characteristics under AC perturbation. (f) The corresponding Nyquist plots derived from impedance spectroscopy. (g) *C*–*V* curves under dark conditions. (h) Zoomed-in Mott–Schottky plots used to extract built-in potential.

Fig. 2a shows the photovoltaic performances such as *V*_OC_, *J*_SC_, FF, and PCE of PSCs as a function of the 2DRP absorber thickness. In the designed PSC, the influence of perovskite thickness ranging from 200 nm to 600 nm is analyzed to investigate the device performance under an operating temperature of 25 °C and an AM1.5G solar irradiance. Fig. 2b demonstrates that the perovskite thickness has a notable effect on the *V*_OC_ and *J*_SC_ at the perovskite thickness above 300 nm. Although a thicker perovskite enhances light absorption, the *V*_OC_ and *J*_SC_ are downshifted with increasing the perovskite layer above 300 nm of the proposed PSCs. The reduction is due to the limited carrier diffusion length of 30 nm at an initial *N*_T_ of 5.0 × 10^16^ cm^−3^. As the perovskite layer becomes much thicker than the carrier diffusion length, photo-induced carriers generated deep within the perovskite experience increased bulk recombination before reaching the charge-transport layers, thereby reducing the *J*_SC_. Similar trends for the *V*_OC_ and *J*_SC_ reduction are also reported in the literature.^32^ The values of FF and PCE reduce with the increment of the perovskite thickness and then are stabilized at the thickness beyond 400 nm (Fig. 2c). The FF of 39.87% and PCE of 6.22% are calculated for the PSC with all 2D interfaces at a 300 nm perovskite layer, respectively. The IPCE profile in Fig. 2d also showed a declined response across visible wavelengths for thicker perovskites, implying incomplete photon harvesting stemming from recombination losses.

As shown in Fig. 2e, the AC measurements showed a suppression in capacitance at thick perovskites due to the inverse dependence of capacitance on the dielectric layer thickness.^33^ The diameter of the semicircular arc in the Nyquist plot represents the *R*_rec_ in the PSC.^34^ Nyquist plots exhibited a considerable reduction in *R*_rec_ with increasing absorber thickness due to higher trap-assisted recombination rates (Fig. 2f). Impedance analysis further supports the FF reduction tendency. Fig. 2g depicts the *C*–*V* characteristics for different perovskite thicknesses in FTO/MoS_2_/2DRP/WS_2_/graphene. As thickness increases, the capacitance curve shifts towards a lower value, suggesting a decrease in the junction's *V*_bi_, as confirmed by Mott–Schottky plots (Fig. 2h). A thinner perovskite achieved a high *V*_bi_ close to 1.2 V, further supporting improved interface quality. In this simulation, the thickness of the 2DRP perovskite is selected to be 300 nm to achieve balanced device performance.

**Fig. 3:**
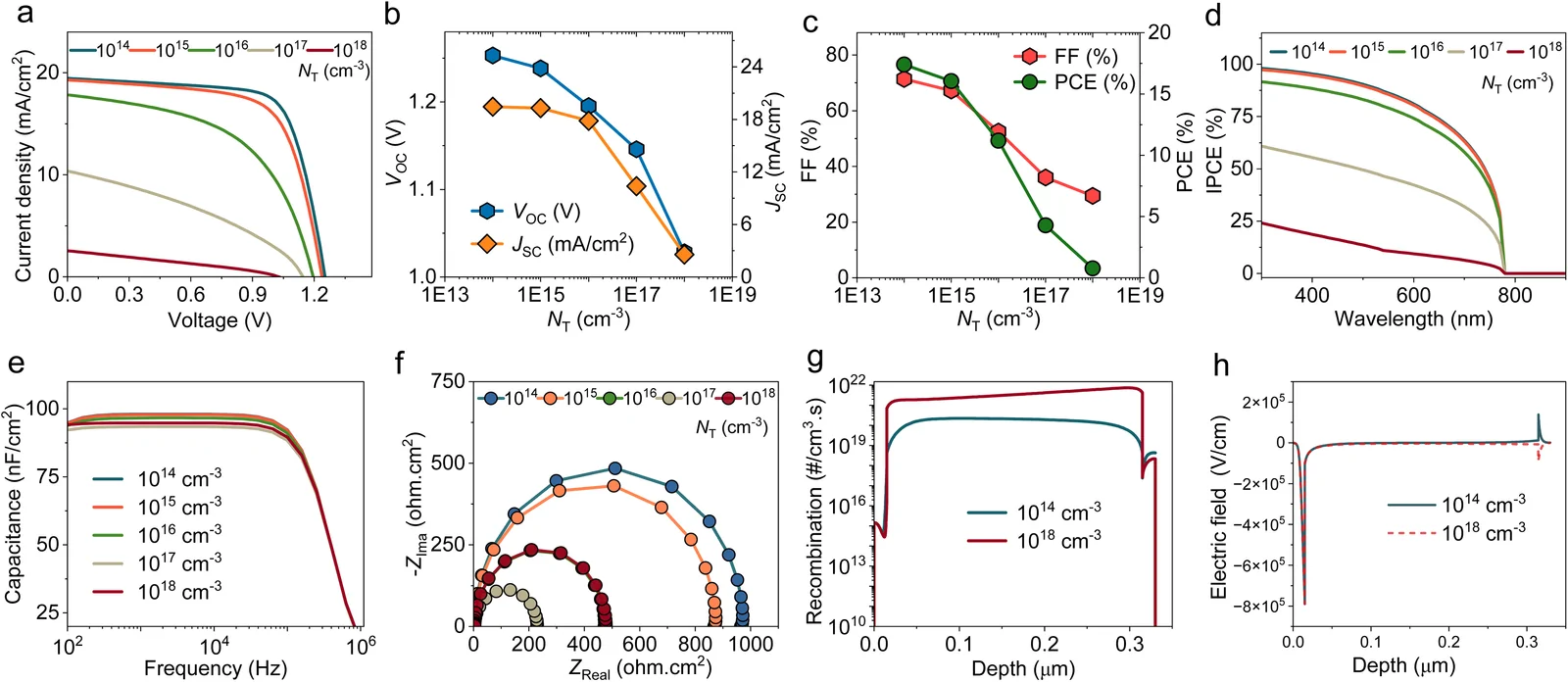
Impact of trap-state density in the perovskite layer on photovoltaic performance metrics of the PSC. (a) *J*–*V* plots. (b) Dependency of *V*_OC_ and *J*_SC_ on perovskite trap states. (c) Evaluation of FF and PCE with trap states. (d) IPCE spectra. (e) *C*–*f* characteristics under AC perturbation. (f) The corresponding Nyquist plots derived from impedance spectroscopy. (g) Recombination profiles at low and high trap-state densities. (h) Electric field analysis.

Fig. 3a shows the PSCs with regard to the alteration in the perovskite's trap-state density (*N*_T_) from 10^14^ to 10^18^ cm^−3^. As exhibited in Fig. 3b and c, an increase in *N*_T_ severely degrades all device parameters, including *V*_OC_, *J*_SC_, FF, and PCE. Defective perovskite captures the electrons generated at the depletion region and recombine with the holes. Therefore, the photogeneration of charge carriers is reduced and recombination rates surge in the PSCs. Subsequently, the movement of photogenerated carriers toward the electrodes is alleviated, affecting the photovoltaic performance of the PSCs.^35^ The IPCE spectra further confirms this trend (Fig. 3d), revealing a notable reduction in quantum efficiency across the broad absorption range for highly defective perovskite.

The *C*–*f* plots in Fig. 3e demonstrate a slight drop in capacitance with increasing *N*_T_, confirming hindered charge-carriers transport. Excessive *N*_T_ shrinks the Nyquist curve (Fig. 3f), indicating reducing *R*_rec_ due to introducing localized energy states operate as recombination bottlenecks, delaying in carrier extraction, thereby degrading FF and *V*_OC_. Additionally, less defective perovskite showed homogeneously distributed recombination across the device, while high trap-state device demonstrating bulk-dominated recombination at the interfaces (Fig. 3g). A lower traps strengthens the electric field at the 2DRP/MoS_2_ interface, as illustrated in Fig. 3h. This facilitates drive more electrons from the perovskite to the ETL while impeding electron leakage, collectively improving device performance.^36^ In the fabricated 2DRP device, a lower *N*_T_ concentration could prolong carrier lifetimes by reducing nonradiative recombination, which will promote charge transport and hence contribute to a higher *V*_OC_ value.^25^ Herein, to optimize the performance of the PSCs, the defect density in the perovskite has been selected to be 10^14^ cm^−3^. The PCE of the PSC at the limited *N*_T_ of 10^14^ cm^−3^ is evaluated to be 17.406%. Accordingly, the charge-carrier diffusion length for 2DRP absorber in the device structure FTO/MoS_2_/2DRP/WS_2_/graphene is 680 nm and, hence the absorber defect concentration must certainly be low to avoid charge-carrier blocking, thus increasing optical losses due to recombination. The carrier diffusion length decreases from 680 nm to 6.8 nm while their lifetime reduces from 10^5^ to 10 ns, when the *N*_T_ of perovskite increase from 10^14^ cm^−3^ to 10^18^ cm^−3^, as listed in Table 3.

**Table 3:** Effect of trap-state density of perovskite on charge carrier properties at a perovskite carrier mobility of 1.78 × 10^−3^ cm^2^ V^−1^ s^−1^

Trap-state density (cm^−3^)	Diffusion length (nm)	Lifetime (ns)
10^14^	680	10^5^
10^15^	210	10^4^
10^16^	68	10^3^
10^17^	21	10^2^
10^18^	6.8	10

**Fig. 4:**
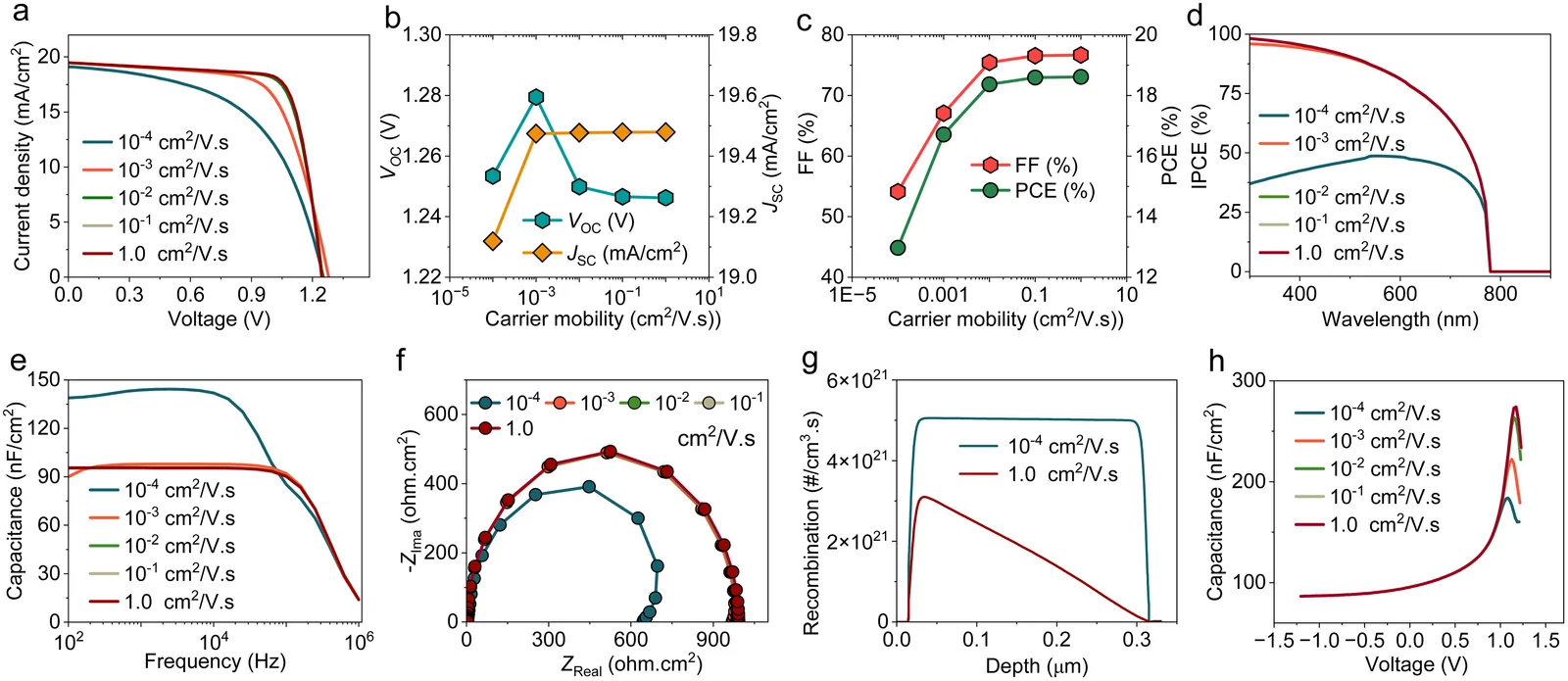
Effect of charge-carrier mobility on the performance of PSCs. (a) *J*–*V* plots. (b) Dependency of *V*_OC_ and *J*_SC_ on charge mobility. (c) Evaluation of FF and PCE with charge mobility. (d) IPCE spectra. (e) *C*–*f* characteristics under AC perturbation. (f) The corresponding Nyquist plots. (g) Recombination rate profiles. (h) *C*–*V* curves under dark conditions.

Fig. 4a exhibits the effect of perovskite carrier mobility on the performance of the PSC while Fig. 4b and c show the corresponding photovoltaic parameters. As the charge mobility of the perovskite increases up to a certain value, the FF and efficiency also rise as the photocarriers are more effectively transferred to the electrodes through the charge transport layers. Further increasing the carrier mobility reaches saturation, and no increase in performance is observed as the maximum number of photocarriers are already collected from the perovskite. For high-mobility device, the IPCE profiles support enhanced photoconversion capacity throughout the visible spectrum (Fig. 4d). As shown in Fig. 4e and f, impedance analysis demonstrates improved *R*_rec_ with higher carrier mobility, affirming mitigated recombination losses. Fig. 4g reveals the recombination rates of PSCs with low and high mobilities. The depth-dependent recombination curves exhibit low variation with carrier mobility. As perovskite carrier mobility decreases (Fig. 4h), the capacitance peak downshifts towards a lower value, suggesting changes in the charge carrier concentration and interface properties of the PSCs.^37^ The optimized device achieves a PCE of 18.613% with an enhanced FF of 76.678%. Experimentally, 1.0 cm^2^ V^−1^ s^−1^ mobility was obtained in 2DRP perovskite with three inorganic layers (*n* = 3), highlighting their potential for efficient charge-carrier transfer.^38^

**Fig. 5:**
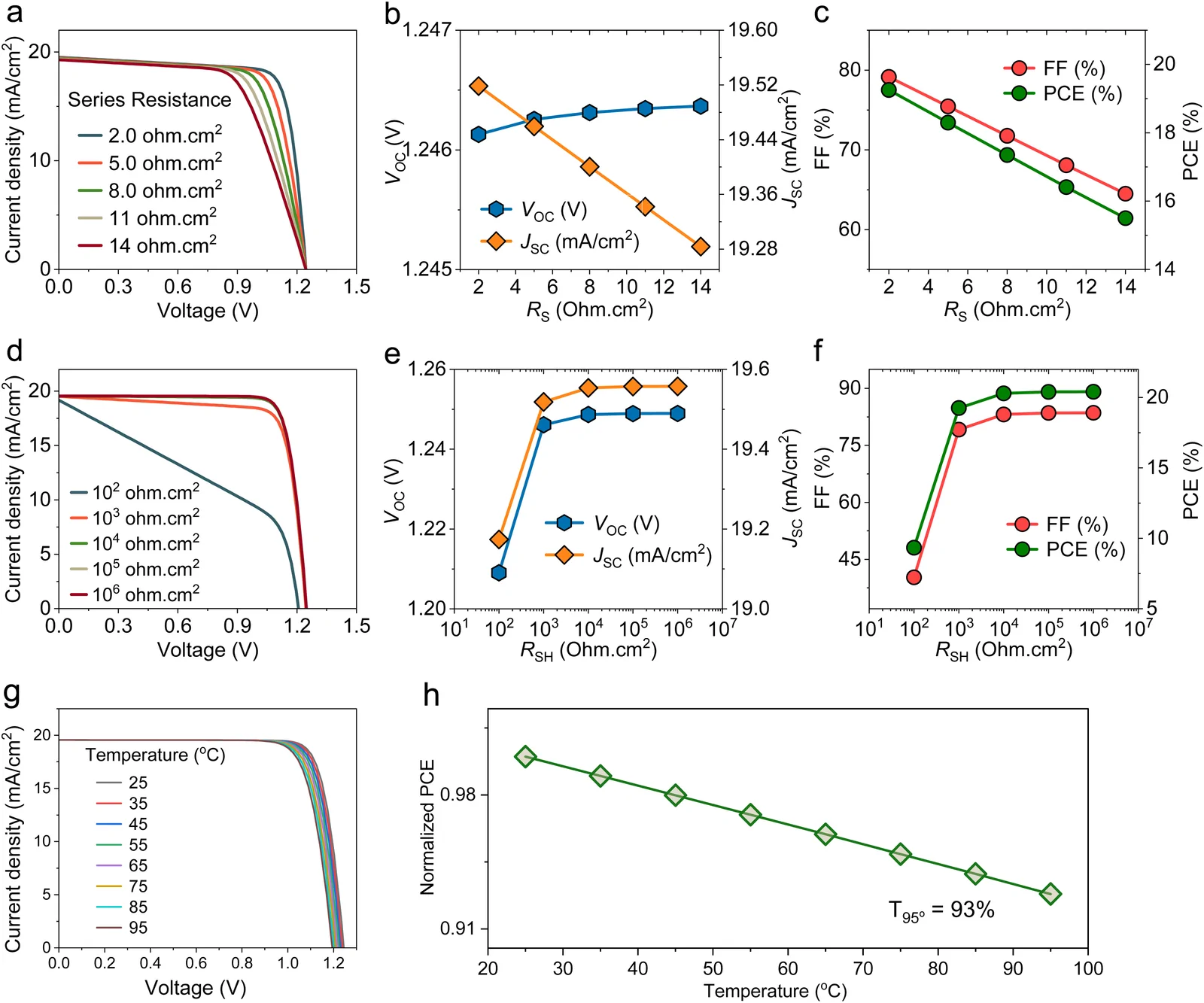
SCAPS-predicted performance reduction of the PSC under varying temperatures and resistance conditions. (a) *J*–*V* plots of devices under different series resistances (*R*_S_). (b) *V*_OC_ and *J*_SC_ evolution in PSCs under varying *R*_S_. (c) FF and PCE varying with *R*_S_. (d) *J*–*V* curves of devices under different shunt resistances (*R*_SH_). (e) *V*_OC_ and *J*_SC_ corresponding to *R*_SH_. (f) *R*_SH_-dependent FF and PCE. (g) *J*–*V* curves of PSCs under different temperatures. (h) Normalized PCE tracking with temperature.

The device behavior is analyzed by changing *R*_S_ from 2.0 to 14 ohm cm^2^ while fixing *R*_SH_ constant at 10^3^ ohm cm^2^. Fig. 5a shows the *J*–*V* plots of PSCs as a function of *R*_S_. Increasing the *R*_S_ has a negligible impact on *V*_OC_ and *J*_SC_ but markedly minimizes FF, as illustrated in Fig. 5b. As a result, device efficiency reduced from 19.249% to 15.501% (Fig. 5c) because of increased ohmic losses that lower carrier extraction. Thereafter, *R*_SH_ varied from 10^2^ to 10^6^ ohm cm^2^, while *R*_S_ is maintained at 2.0 ohm cm^2^ and the corresponding *J*–*V* curves are revealed in Fig. 5d. As observed, increasing the *R*_SH_ strongly improves all photovoltaic parameters (Fig. 5e and f), implying reduced leakage pathways that promote recombination rate. To probe the effect of temperature on the performance of the PSC, the temperature-dependent device performance was performed with varied from 25 °C to 95 °C. Fig. 5g exhibits that as the temperature increases, the FF and *V*_OC_ of all the PSCs drop, causing a downshifting in the PCE. This behavior is attributed to enhanced thermally activated carrier recombination and changes in the electrical transport properties incorporated in the SCAPS-1D model.^39^ As shown in Fig. 5h, normalized PCE is gradually reduced with increasing operating temperature, and the optimized device maintained 93% of its original performance at 95 °C.

**Fig. 6:**
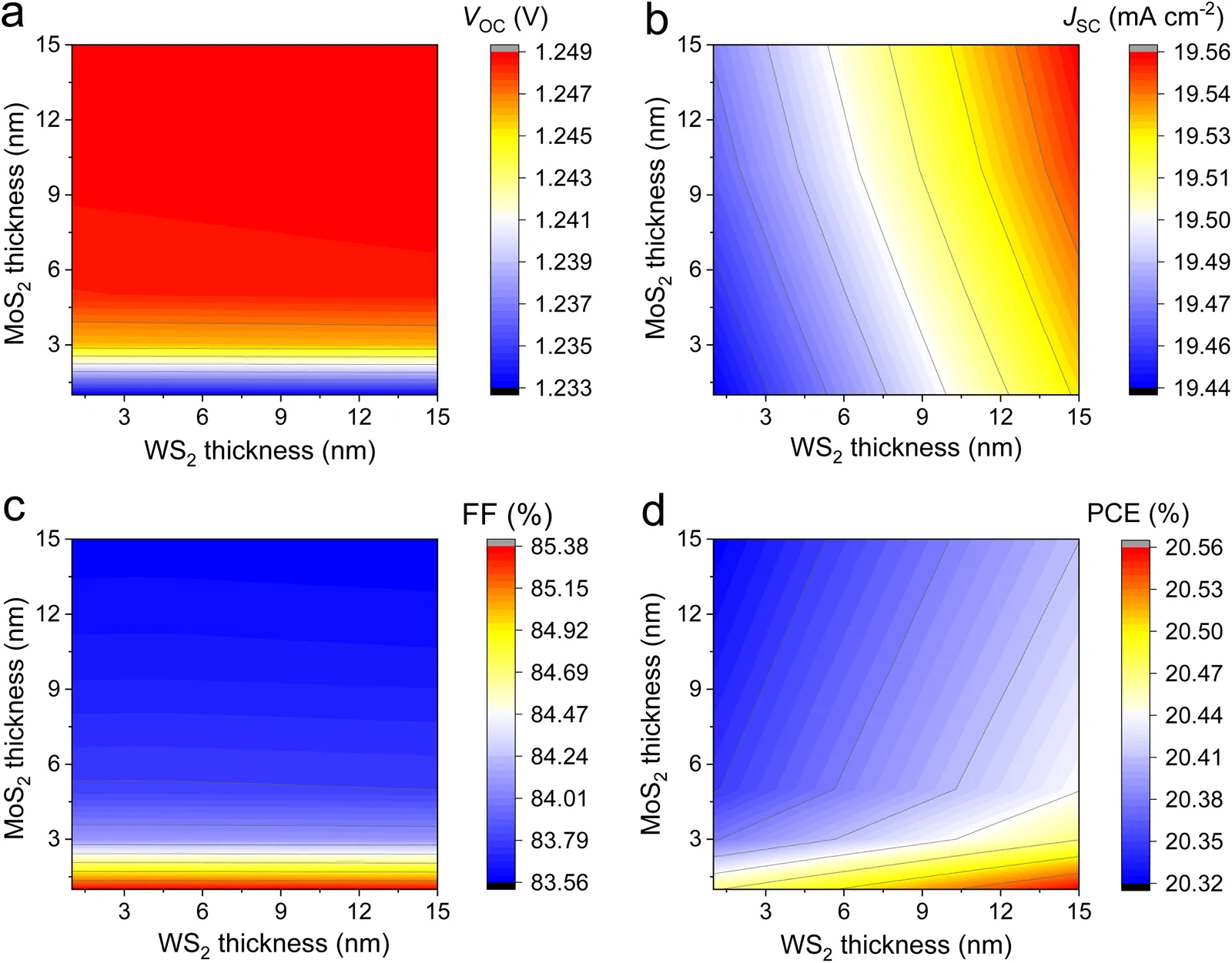
Evaluation the effects of the thickness of charge transporting layers on RP perovskite solar cells. Contour plots for (a) *V*_OC_, (b) *J*_SC_, (c) FF, and (d) PCE.

Finally, the thicknesses of the WS_2_ and MoS_2_ layers were concurrently varied in a range of 1–15 nm while keeping other parameters constant in order to analyze the effect of charge-transporting layer thicknesses on device performance. Fig. 6 exhibits contour plots of photovoltaic parameters as a function of layer thickness. As observed, the simulated PSC showed a marginal sensitivity to the WS_2_ and MoS_2_ thicknesses. Specifically, the efficiency changed by less than 0.3%, while the changes in *V*_OC_ and *J*_SC_ were negligible. A slight improvement in FF was revealed with increasing MoS_2_ thickness, which may be associated with an increase in ETL resistance. These results indicate that the photovoltaic performance is largely insensitive to the thicknesses of the ultrathin TMD transport layers within the investigated range.

**Fig. 7:**
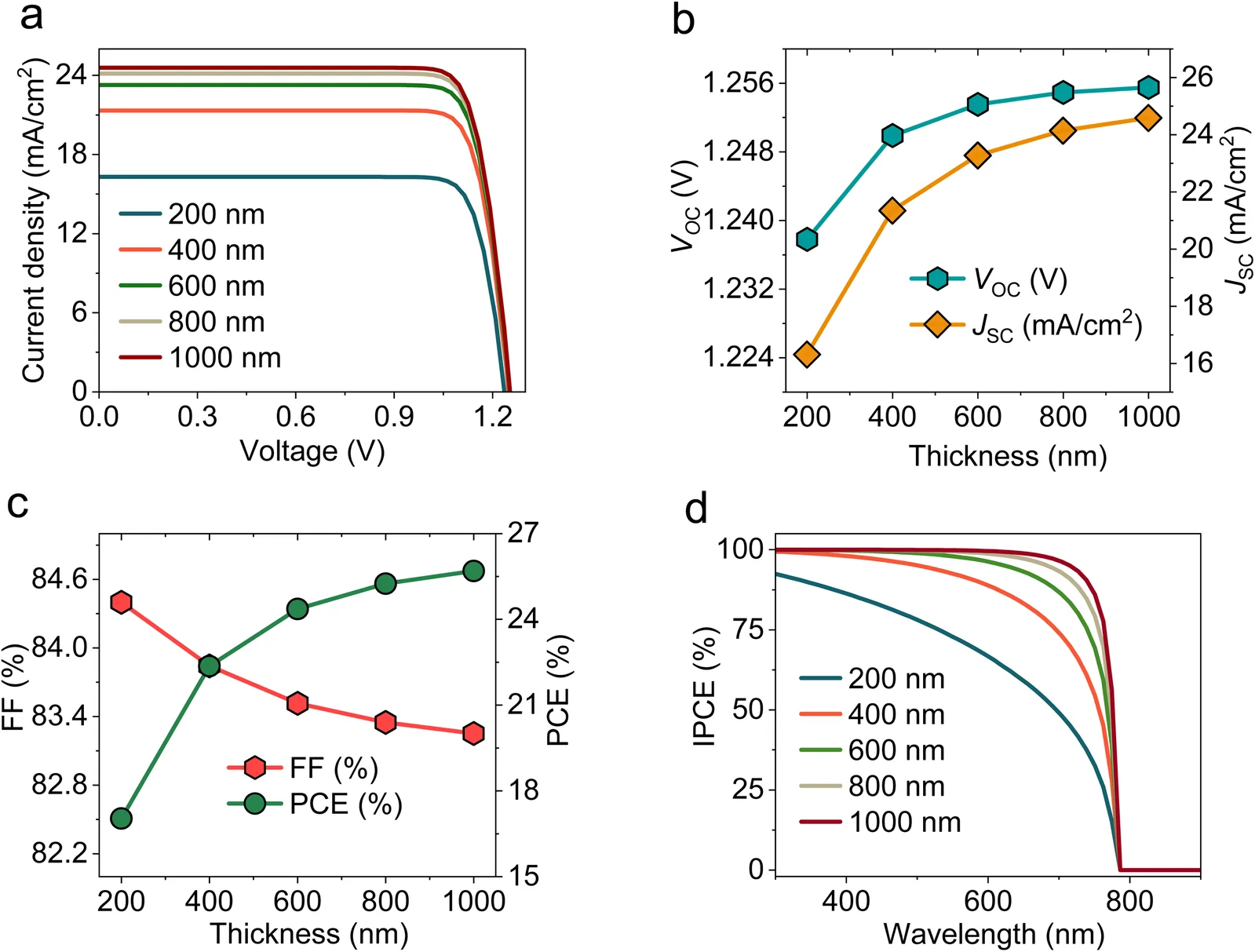
Re-optimization of the 2D Ruddlesden–Popper perovskite absorber thickness under the final optimized device parameters. (a) *J*–*V* plots. (b) *V*_OC_ and *J*_SC_ evolution. (c) FF and PCE varying with perovskite thickness. (d) IPCE spectra.

Fig. 7a shows the re-simulated *J*–*V* characteristics of RP devices with different perovskite thicknesses under the final optimized device parameters. As shown in Fig. 7b and c, *J*_SC_ and PCE parameters improve rapidly with increasing perovskite thickness from 200 nm to 800 nm, then begin to saturate beyond 800 nm. Also, IPCE also reaches near saturation around 800 nm, indicating that most incident photons are already being absorbed, as depicted in Fig. 7d. However, FF declines slightly with increasing thickness, indicating increased carrier transport resistance in thicker perovskites. The optimized device attained a simulated PCE of 25.249% with a FF of 83.348%, a *V*_OC_ of 1.254 V, and a *J*_SC_ of 24.14 mA cm^−2^ at a perovskite thickness of 800 nm.

**Fig. 8:**
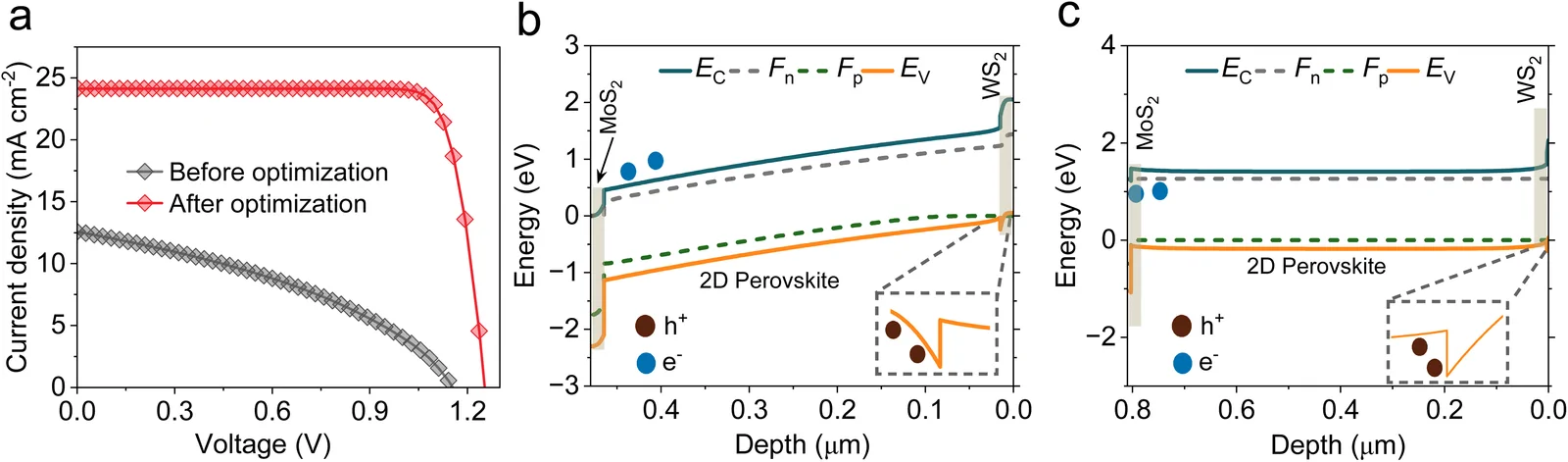
The device performance and energy band alignment of 2D PSCs before and after optimization. (a) *J*–*V* plots. (b) Band diagram structure before optimization. (c) Band diagram structure after optimization.

Fig. 8a shows the *J*–*V* curves of the simulated PSCs before and after optimization. The optimized device revealed a significant enhancement in the *J*_SC_ parameter because more photons are absorbed in the perovskite layer, thus improving carrier generation. In addition, the optimized device possesses lower defect density, which allows more photogenerated carriers to reach electrodes before recombining inside the absorber layer, further enhancing FF and *J*_SC_. Decreased recombination sites increase the separation of charge carriers, hence improving quasi-Fermi level splitting (QFLS) and *V*_OC_. Fig. 8b and c depict the band diagram structures of the simulated devices under illumination conditions at constant bandgap and electron affinity of the constituent materials. The conduction band offset (CBO) and valence band offset (VBO) at the MoS_2_/2DRP and 2DRP/WS_2_ interfaces are slightly reduced after device optimization. The small variations in CBO and VBO are mainly due to changes of the electrostatic potential, band bending, and QFLS that correlate with the modified transport and recombination conditions.

As illustrated in Table 4, the experimentally demonstrated 2DRP PSCs obtained PCEs in the range of 15–21%, and many theoretical studies predicted efficiencies above 22%. Thus, the simulated PCE of 25.249% is an improvement over the reported values in this comparison. The proposed FTO/MoS_2_/2DRP/WS_2_/graphene configuration presents a mainly all-2D platform in comparison with conventional PSC architectures with 3D perovskites, metal-oxide charge-transport layers, and metallic electrodes. The ultrathin MoS_2_ and WS_2_ TMDs offer compact interfaces with the RP perovskite and can enable selective electron and hole extraction, respectively. In contrast, the 2D RP perovskite offers improved environmental stability and possibilities of dimensional and electronic-structure engineering. The synergic combination of these materials opens up systematic engineering of carrier-selective interfaces and can possibly mitigate interfacial recombination losses.

**Table 4:** Comparison of the device performance for the present work with closely related 2DRP-based PSCs

Year	Study type	Perovskite	*V*_OC_ (V)	*J*_SC_ (mA cm^−2^)	FF (%)	PCE (%)	Ref.
2018	Experimental	(ThMA)_2_(MA)_2_Pb_3_I_10_	1.07	18.89	76.30	15.42	25
2019	Experimental	(4FPEA)_2_(MA)_4_Pb_5_I_16_	1.16	19.00	79.00	17.30	40
2020	Experimental	(ThFA)_2_(MA)_2_Pb_3_I_10_	1.05	20.17	79.00	16.72	41
2022	Experimental	(4FPEA)_2_(FA)_4_Pb_5_I_16_	1.18	22.45	79.53	21.07	42
2023	Simulation	(ThMA)_2_(MA)_2_Cu_3_I_10_	1.06	24.05	80.00	22.97	43
2026	Simulation	(MTEA)_2_(MA)_4_Pb_5_I_16_	1.49	17.17	81.03	20.79	44
2026	Simulation	(ThMA)_2_(MA)_2_Pb_3_I_10_	1.25	24.14	83.34	25.24	This work

## 4. Conclusion

In summary, we systematically evaluated the photoelectric performance of a novel 2D-layered solar cell with a 2D Ruddlesden–Popper perovskite absorber. Desirable WS_2_ and MoS_2_ are proposed as 2D photoelectrodes in the PSCs as HTL and ETL, respectively. The device band structure showed proper energy-level alignment across all layers with enhanced charge-carrier transport. SCAPS-1D simulations showed that increasing carrier mobility of the 2DRP perovskite enhanced charge transport and influenced the capacitance characteristics predicted by the drift-diffusion model. Additionally, it is exhibited that the tuning of defect density can reduce the localization of recombination sites and increase carrier diffusion length, thereby improving the overall device efficiency. The thickness of the 2D perovskite is optimized at 800 nm to achieve balanced photoconversion efficiency. Increasing series resistance led to significant dropping in FF and PCE by limiting current flow, while higher shunt resistance efficiently mitigated leakage currents, thus boosting PSC performance. An additional transport-layer thickness sensitivity analysis demonstrated that varying the MoS_2_ and WS_2_ thicknesses induced marginal changes in the device performance, suggesting that the proposed PSC is largely insensitive to the thickness of the ultrathin TMD transport layers. Accordingly, the efficiency of the designed 2D PSC is simulated to be 25.249% with a FF of 83.348%, a *V*_OC_ of 1.254 V, and a *J*_SC_ of 24.14 mA cm^−2^. Our numerical calculations can guide experimental researchers in improving photovoltaic performance of 2DRP perovskite by identifying suitable absorber thickness, enhancing crystallinity and defect passivation, and improving charge transport dynamics based on the proposed device structure.

## Conflicts of interest

The authors declare no conflicts of interest.

## Data Availability

The data will be available from the corresponding author on reasonable request.
